# Ophthalmic Solutions with a Broad Antiviral Action: Evaluation of Their Potential against Ocular Herpetic Infections

**DOI:** 10.3390/microorganisms10091728

**Published:** 2022-08-27

**Authors:** Carla Zannella, Annalisa Chianese, Maddalena De Bernardo, Veronica Folliero, Francesco Petrillo, Anna De Filippis, Giovanni Boccia, Gianluigi Franci, Nicola Rosa, Massimiliano Galdiero

**Affiliations:** 1 Department of Experimental Medicine, University of Campania “Luigi Vanvitelli”, 80138 Naples, Italy; 2 Department of Medicine, Surgery and Dentistry “Scuola Medica Salernitana”, University of Salerno, 84081 Baronissi, Italy; 3 Department of Ophthalmology, University of Catania, 95123 Catania, Italy

**Keywords:** ophthalmic solutions, ocular, antiviral activity, HSV-1, eye, keratitis

## Abstract

HSV-1 can be associated with severe and recurrent eye infections characterized by a strong inflammatory response that leads to blepharoconjunctivitis, epithelial and stromal keratitis, and retinal necrosis. The incidence of HSV-1 keratitis is 1.5 million every year worldwide, including more than 40,000 new cases exhibiting serious visual failures. Generally, the therapy uses antiviral drugs to promote healing; however, there are currently no compounds that are able to completely eradicate the virus. In addition, the phenomenon of resistance is rapidly spreading among HSV-1 strains, creating mutants developing resistance to the common antiviral drugs; therefore, deep research on this issue is warranted. The efficacy of different ophthalmic solutions already on the market was evaluated for reducing HSV-1 infection. Different plaque assays were set up on epithelial cells, revealing that two ophthalmic solutions were able to inhibit viral replication in the early stages of infection. The data were further confirmed by molecular tests analyzing the expression levels of the principal genes involved in HSV-1 infection, and a strong reduction was observed after only 1 min of eye-drop treatment. Collectively, these results suggested the use of ophthalmic solutions as potential antiviral options for the treatment of ocular herpetic infection.

## 1. Introduction

Herpes simplex virus type 1 (HSV-1) is the aetiologic agent of herpes labialis, a very common infection spreading all over the mouth and characterized by small, but burning sores. A last report, the World Health Organization (WHO) declared that more than 67% of the global population has been infected by HSV-1, especially during childhood [1]. Primary infection occurs by direct contact with the secretions of an infected individual. In most cases, it is subclinical, or causes mild pseudo-influenza symptoms. From the site of primary infection, the virus, migrating along the sensitive nerve endings, reaches the sensory nerve ganglia, where it remains in a latent state within the nucleus of the neurons [2]. The reactivation from the latency state can be triggered by some local (lesions close to the areas innervated by infected neurons) or general stimuli (stress, fever, hormonal changes), which determine a new migration of the virus from the neuron towards the surface innervated by the nerve [3,4]. Therefore, during a reactivation event, the virus can manifest itself in the original site of the infection, or in any other site innervated by the infected ganglion. Reactivation lasts several days, after which the virus reverts to the latent state [5].

Generally, the infection is asymptomatic or paucisymptomatic, but rarely, it can lead to severe and even fatal manifestations [6,7,8,9]. In fact, among immunocompromised individuals, infections such as herpes simplex keratitis are quite widespread and are often accompanied by serious complications, such as perforation of the cornea and/or blindness, if left unchecked and untreated [10,11,12,13]. Herpes simplex keratitis represents the leading cause of corneal ulcers and blindness in the world. Its incidence reaches 1.5 million cases every year worldwide, including more than 40,000 new cases, causing serious visual damage [14]. HSV-1 can cause various ocular manifestations, as it is able to affect every eye district. The eye can be involved during primary herpes infection, or more commonly, during its reactivation. Primary ocular infections generally manifest with vesicular skin lesions, blepharitis, and conjunctivitis, while the main manifestations of recurrent ocular herpetic diseases are epithelial keratitis, stromal keratitis, endotheliitis, and uveitis [15]. Epithelial keratitis accounts for 60% of ocular HSV-1 cases and is characterized by punctate keratitis, evolving towards a dendritic form and geographic ulcer. The main symptoms are eye pain, photophobia, redness, and blurred vision [15,16]. Stromal keratitis represents 20–48% of recurrent ocular herpetic cases, and it is the disease most correlated with decreased vision due to corneal scarring and neovascularization, which increase with each recurrence of the infection [17,18].

Antiviral therapy is used to speed up healing and reduce the extent of the disease [19,20]. The therapeutic approach is mainly based on the use of topical or systemic antivirals for the epithelial keratitis, with the addition of topical cortisones for the stromal disease [21]. However, current therapeutic strategies are not able to eradicate the virus from its latent state and are unable to completely block the inflammatory phenomena underlying corneal scarring, with consequent loss of vision. In addition, another of the main consequences of herpes simplex keratitis is the damage to the corneal innervation, resulting in neurotrophic keratopathy [22]. The latter causes an important alteration of the ocular surface, with consequent lacrimal dysfunction and dry eye. Long-term therapy with topical antiviral drugs helps to control the disease and prevents disease-relapse, but, because of its toxicity, it can further damage the ocular surface. In addition, the phenomenon of resistance is rapidly diffusing, creating mutant strains that develop resistance to the conventional antiviral drugs [23,24,25,26,27]. For all these reasons, the aim of the present study was to search for new antiviral approaches capable of counteracting ocular HSV-1 infections. We tested, for the first time, the in vitro efficacy of four ophthalmic solutions—Iodim, Ozodrop, Dropsept, and Septavis—regarding the inhibition of HSV-1 replication. The four eye drops are solutions already on the market used as adjuvants for the cleaning, protection, and repair of the corneal epithelium. However, several studies reported their additional inhibitory effect against human pathogens, including viruses, bacteria, fungi, and amoebas, recognizing that the eye drops could be effective in both preventing and/or controlling different infectious diseases of the ocular surface [28,29,30,31,32].

## 2. Materials and Methods

### 2.1. Test Compounds

The composition of Ozodrop (FB Vision, Ascoli Piceno, Italy), Dropsept (IROMED group srl, Rome, Italy), Septavis (MEDIVIS, Catania, Italy) and Iodim (MEDIVIS, Catania, Italy) eye drops has been previously specified [33].

### 2.2. Cell and Virus Culture

Vero cells (ATCC CCL-81, Manassas, VA, USA) were grown in Dulbecco’s Modified Eagle Medium (DMEM) with 4.5 g/L glucose supplemented with 2 mM L-Glutamine, 100 IU/mL penicillin-streptomycin solution, and 10% Fetal Bovine Serum (FBS). All the materials used for cell culture were acquired from Microtech srl, Naples, Italy. HSV-1 (strain SC16), containing a lacZ gene driven by the cytomegalovirus IE-1 promoter to express beta-galactosidase, was cultured in Vero cells, as previously reported [34].

### 2.3. Antiviral Activity

The effect of ophthalmic solutions on HSV-1 infection was evaluated in different plaque reduction assays. These assays differ for the time in which the compound has been added to the cells, during the infection (co-treatment), following the incubation of the compound-virus (virus pre-treatment), prior the viral infection (cell pre-treatment), and finally, after the viral infection (post-treatment). In detail, in the co-treatment assay, each eye-drop and virus at 0.01 multiplicity of infection (MOI) was incubated on a Vero cell monolayer (5 × 10^5^ cells/well) for different periods of incubation (15 s, 30 s, 1 min, 10 min, 30 min, and 1 h) at different volumes (100, 50, 25, and 12.5 µL). In the virus pre-treatment test, each eye-drop was combined with the virus at 0.1 MOI, with the same conditions previously mentioned, and then the mixture was titrated on the cell monolayer; in the cell pre-treatment assay, Vero cells were pre-treated with each ophthalmic solution and then infected with HSV-1 at 0.01 MOI. Finally, in the post-treatment test, cells were first infected, and then treated with each ophthalmic solution at different periods of incubation, as in the previous tests. In all treatments, at the end of each treatment, the cell monolayer was first washed with citrate buffer (pH 3, for 5 min) and then overlaid with carboxymethylcellulose (CMC) 5% in the presence of the culture medium. After 48 h, Vero cells were fixed and stained with 4% formaldehyde and 0.5% crystal violet, respectively, and the viral plaques were counted. The viral titer was expressed as PFU/mL (Log 10) compared to untreated HSV-1 infected cells (ctr-), considering that ctr- corresponds to 2 × 10^3^ PFU/mL. The experiments were performed in triplicate. For each treatment, different reference compounds were used as a positive control (ctr+): melittin (for the co-treatment and virus pre-treatment assay), dextran sulfate (for the cell pre-treatment assay), and acyclovir (for the post-treatment assay) [35]. In detail, melittin and dextran sulfate inhibit the early phases of herpetic infection, but in different modes. The former is active on the viral envelope; meanwhile, the latter is active on the cell membrane. Finally, acyclovir is a drug with well-reported abilities to block HSV-1 replication [35].

### 2.4. Real-Time PCR

The virus pre-treatment assay was chosen for the molecular test and performed as previously reported at the same volumes (from 100 to 12.5 µL), and after only 1 h stimulation. After 48 hpi, RNA was extracted by using the TRIzol reagent (Thermo Fisher, Waltham, MA, USA). Total RNA was quantified through the absorbance at 260 nm (NanoDrop 2000, Thermo Fisher Scientific, Waltham, MA, USA) and retrotranscribed to cDNA by 5× All-In-One RT Master Mix (Applied Biological Materials, Richmond, VA, Canada). Real-time PCR was performed using a CFX thermal cycler (Bio-Rad, Hercules, CA, USA) and amplified through BrightGreen 2× qPCR MasterMix-No Dye (Applied Biological Materials, Richmond, VA, Canada) and 0.1 µM of primer. The relative target threshold cycle (Ct) values of UL54 (immediate early gene), UL52 (early gene), and UL27 (late gene) were normalized using glyceraldehyde 3 phosphate dehydrogenase (GAPDH) as the housekeeping gene. The mRNA levels of cells treated with ophthalmic solutions were calculated via the 2^−∆∆Ct^ method. The primer sequences and the thermal conditions are shown in Table 1.

### 2.5. Statistical Analysis

All tests were carried out in triplicate and indicated as mean ± standard deviation (SD), as evaluated by GraphPad Prism. Statistical differences were calculated via two-way ANOVA, followed by a Bonferroni post hoc test; a value of *p* ≤ 0.05 was considered significant.

## 3. Results and Discussion

### 3.1. Antiviral Activity against HSV-1

**Figure 1:**
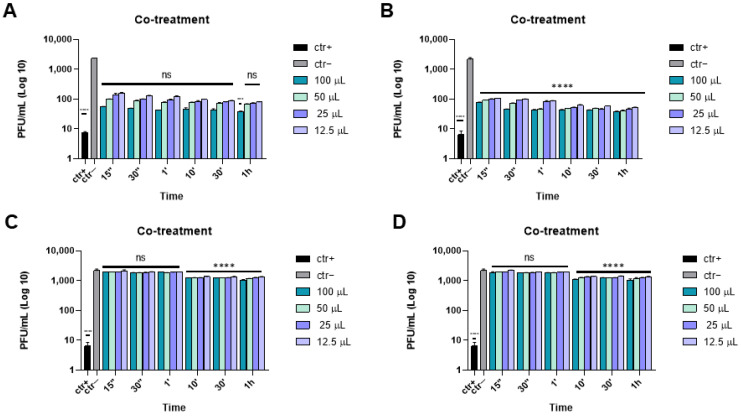
Co-treatment assay of the antiviral activity of ophthalmic solutions against HSV-1 at different times of incubation. Iodim (**A**), Ozodrop (**B**), Dropsept (**C**), and Septavis (**D**) were incubated simultaneously with HSV-1 on Vero cells. Melittin [35] was used as a positive control (ctr+). Data are means of three independent experiments. Statistical analyses were determined by two-way ANOVA, followed by a Bonferroni post hoc test. Significance refers to the negative control (ctr–), i.e., the infected cells. **** *p* < 0.0001; ns: non-significant.

We previously reported the antiviral activity of the same eye drops against the causative agent of the current pandemic, i.e., COVID-19 [33]. According to the previous data, Iodim, Ozodrop, Dropsept, and Septavis did not show any toxicity at any time during the incubation on the cellular model used during the in vitro experiments [33]. Next, we decided to investigate the effect of the eye drops on the inhibition of the HSV-1 infection. First, a co-treatment assay was performed to investigate the ability of ophthalmic solutions to interfere, in general, with the herpes replication cycle. HSV-1 and each eye drop at the indicated volumes (100, 50, 25, and 12.5 µL) or melittin (as a reference compound) were incubated simultaneously on a Vero cell monolayer. Several incubation times from 15 s to 1 h were used for our experimental design, since we tried to mimic the real condition of application of the ocular solution. Two eye drops, Iodim and Ozodrop, were very effective in inhibiting viral replication at all incubation times tested (Figure 1A,B). As reported, the solutions exhibited a very similar dose–dependent inhibition, with a half maximal inhibitory concentration (IC50 in Table 2) at 30 µL at the time of 15 s; after 30 s, the IC50 reduced to 25 µL, and it was less than 12.5 µL for the other tested times (1 min, 10 min, 30 min, and 1 h). This evidence indicated that little more than one drop (<12.5 µL) of both solutions was able to halve the herpetic infection, starting from only 1 min of treatment. On the contrary, Dropsept and Septavis were not active against HSV-1 (Figure 1C,D).

**Figure 2:**
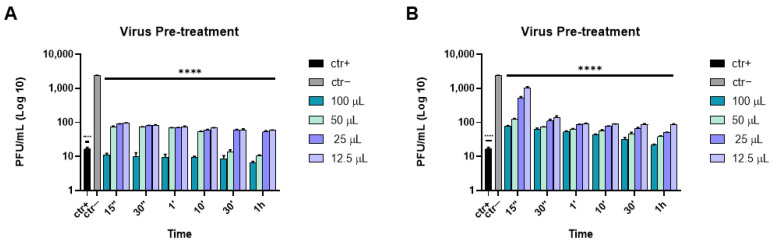
Virus pre-treatment assay of the antiviral activity of ophthalmic solutions against HSV-1 at different times of incubation. Iodim (**A**) and Ozodrop (**B**) were first incubated with HSV-1, and then the mixture was used to infect Vero cells. Melittin [35] was used as a positive control (ctr+). Data are means of three independent experiments. Statistical analyses were determined by two-way ANOVA, followed by a Bonferroni post hoc test. Significance refers to the negative control (ctr–), i.e., the infected cells. **** *p* < 0.0001.

Subsequently, we tried to clarify the mechanism of action of these two active solutions. Thus, to assess whether these solutions could interact with the viral particles or with the cell surface, or could inhibit the viral replication, virus pre-treatment, cell pre-treatment and post-treatment assays were conducted, respectively. In the former, HSV-1 and different volumes of each solution or melittin (as reference compound) were first incubated for the same times described above at 37 °C; then the mixture was diluted and inoculated on the Vero cell monolayer. In this case, Iodim and Ozodrop also showed an inhibitory activity against HSV-1 (Figure 2). In detail, the Iodim antiviral effect was considerably improved, as observed by its high inhibitory action at 100 µL, for all incubation times, and at 50 µL after the longer time of incubation (1 h). Moreover, the IC50 dropped at 12.5 µL (Table 2), ranging from 30 s to 1 h of stimulation. However, Ozodrop showed a reduced inhibition efficacy compared to Iodim, with an IC50 at 75 µL at 15 s, 25 µL at 30 s, and less than 12.5 µL from 1 min to 1 h of stimulation (Table 2). The results suggested that both of these eye drops were able to interact with the viral membrane. We assume that the ophthalmic solutions act as detergents by destroying the viral surface and interfering with all the subsequent events, including the viral attachment and entry in the host cells.

To identify the best eye drop and the amount of it exhibited the higher effectiveness, it was important to establish that the antiviral activities observed could be useful at volumes that were possible to achieve without inducing toxic effects on the cells. Therefore, the relative effectiveness of the two ophthalmic solutions in inhibiting viral replication was compared to cell viability (CC50 value/IC50 value) to obtain a therapeutic index (TI) (Table 1). Iodim was determined to be the best eye drop, with a TI of 8, and it was able to consistently and rapidly reduce the HSV-1 infection with only 12.5 µL and after just 30 s.

**Figure 3:**
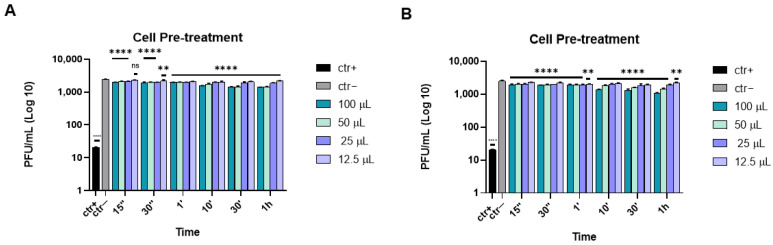
Cell pre-treatment assay of the antiviral activity of ophthalmic solutions against HSV-1 at different times of incubation. Iodim (**A**) and Ozodrop (**B**) were first incubated on the cell monolayer prior to infection. Dextran-sulphate [35] was used as a positive control (ctr+). Data are means of three independent experiments. Statistical analyses were determined by two-way ANOVA, followed by a Bonferroni postdhoc test. Significance refers to the negative control (ctr–), i.e., the infected cells. ** *p* < 0.001; **** *p* < 0.0001; ns: non-significant.

To exclude other types of interactions retained by the two eye drops, further tests were carried out. In the cell pre-treatment assay, Vero cells were incubated with either the compounds or dextran sulfate (as reference compound) at different volumes and times at 37 °C, and then the cell monolayer was infected with HSV-1. Figure 3 shows that neither Iodim and Ozodrop were able to interact with the target cell.

**Figure 4:**
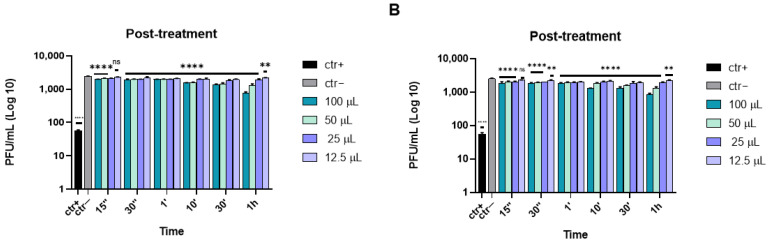
Post-treatment assay of the antiviral activity of ophthalmic solutions against HSV-1 at different times of incubation. Cells were first infected with HSV-1 and then treated with Iodim (**A**) or Ozodrop (**B**). Acyclovir [35] was used as a positive control (ctr+). Data are means of three independent experiments. Statistical analyses were determined by two-way ANOVA, followed by a Bonferroni post hoc test. Significance refers to the negative control (ctr–), i.e., the infected cells. ** *p* < 0.001; **** *p* < 0.0001; ns: non-significant.

Finally, in the post-treatment assay, the cells were first infected with HSV-1 and then treated with different volumes of either the eye drops or acyclovir (as a reference compound). In this case, the two compounds did not exhibit any inhibitory efficacy, confirming that they did not act inside the cell at the viral replication phase (Figure 4).

Collectively, these data indicate that Iodim and Ozodrop impact the early stages of HSV-1 infection, likely by directly damaging the viral membrane, as suggested by the virus pre-treatment results. This evidence highlights the potential therapeutic use of the two eye drops. In addition to serving as cleaning and protection solutions for the ocular surface, they could also interfere with harmful agents, such as HSV-1, preventing them from being able to infect the eye.

### 3.2. Real-Time PCR

**Figure 5:**
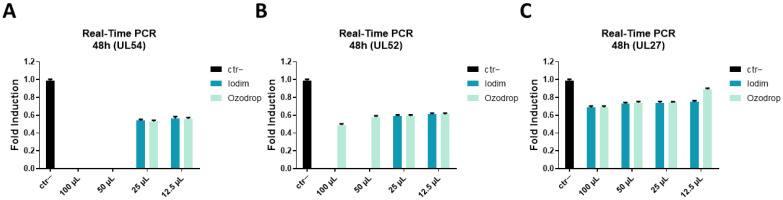
Real-time PCR. A molecular assay was performed to evaluate the effect of ophthalmic solutions on the viral gene expression. Iodim and Ozodrop were tested at different volumes at 48 hpi. The expression of UL54 (**A**), UL52 (**B**), and UL27 (**C**) was analyzed. Data are means of three independent experiments. Ctr- refers to infected, but not treated cells.

Molecular testing using real-time PCR was conducted to confirm the data obtained in vitro through the plaque assays. The virus pre-treatment assay was carried out by treating HSV-1 particles with the two active solutions for 1 h at the same volumes, as described above. Then, the mixture was diluted on Vero cell monolayers, and viral RNA was extracted at 48 h post-infection (hpi) and reverse-transcribed into cDNA. The expression of the immediate early (IE, UL54), early (E, UL52), and late (L, UL27) genes was evaluated. The IE UL54 gene codes for the HSV-infected cell culture polypeptide 27 (ICP27) protein involved in all stages of viral mRNA biogenesis from transcription, RNA processing, and export to translation machinery [36,37]. This protein acts as a multifunctional regulator by controlling all the subsequent steps of viral gene expression [37]. The E UL52 gene participates in the production of the helicase/primase complex, necessary for DNA unwinding at the replication fork and the synthesis of primers during virus replication [38,39]. Finally, the L UL27 gene encodes HSV glycoprotein B (gB), the fusion protein involved in the viral entry into the host cell [40,41,42]. As reported in Figure 5, Iodim and Ozodrop reduced the expression of all three viral genes. In detail, Iodim was able to totally block UL54 and UL52 expression after 48 h, both at 100 and 50 µL. On the other hand, Ozodrop completely interfered with UL54 gene expression, but only reduced UL52 transcription. The expression of the late gene UL27 was not greatly influenced 48 hpi. Moreover, in this case, the effect of Iodim was greater than the effect of Ozodrop, which is very similar to that we observed via the plaque assays. These data confirmed that the ophthalmic solutions could act early during the infection process by preventing and blocking the entry of the virus, especially interfering with IE and E gene expression.

## 4. Conclusions

The WHO has estimated that approximately 67% of the global population is affected by the infection caused by HSV-1. It can cause serious ocular damage, such as keratitis, uveitis, and retinitis [43,44,45]; thus, it is essential to try to limit infection and increase prevention to reduce the spread of the virus. The aim of the present study was to provide disinfectant solutions capable of restricting the ocular transmission of HSV-1. In this regard, four eye drop solutions (Iodim, Ozodrop, Dropsept, and Septavis), already on marketed, were tested in order to estimate their effectiveness against herpetic infection. Several studies reported their antimicrobial efficacy, both in vivo and in vitro. Previous evidence demonstrated the efficacy of Iodim against Human immunodeficiency virus type 1 (HIV-1), influenza A virus, and SARS-CoV-2 [33,46,47,48,49,50,51]. Furthermore, its antibacterial and antifungal activity was tested, showing a strong inhibition after 5 min and 24 h of incubation [28,52,53,54]. Ozodrop also showed a remarkable antifungal (against Candida), antibacterial (against *Staphylococcus aureus* and *Pseudomonas aeruginosa*) and antiviral (against SARS-CoV-2) activity [55,56,57,58,59,60,61,62,63,64]. Dropsept was found to be effective in the treatment of keratitis caused by *Acanthamoeba*, and only recently, its antiviral activity against SARS-CoV-2 has been reported, with an inhibitory potential of 77% at 50 µL after 15 s. Our data are in accordance with the literature reporting a dose–dependent reduction in HSV-1 infection through in vitro studies. Iodim and Ozodrop showed an inhibitory action in the early phase of the viral attack, likely by damaging the viral membrane. On the contrary, Dropsept and Septavis exhibited no inhibitory effect. Collectively, these data indicated that Iodim and Ozodrop could be used as antiviral options in the early stages of HSV infection, by restricting the herpetic infection after only 1 min of contact with the virus.

## Figures and Tables

**Table 1:** Primer sequences and thermal conditions used for real-time PCR.

Gene	Sequence
UL54	Forward: TGGCGGACATTAAGGACATTG Reverse: TGGCCGTCAACTCGCAG
UL52	Forward: GACCGACGGGTGCGTTATT Reverse: GAAGGAGTCGCCATTTAGCC
UL27	Forward: GCCTTCTTCGCCTTTCGC Reverse: CGCTCGTGCCCTTCTTCTT
GAPDH	Forward: CCTTTCATTGAGCTCCAT Reverse: CGTACATGGGAGCGTC
**Thermocycler conditions**	
95 °C for 10 min	
95 °C for 15 s 60 °C for 1 min 72 °C for 20 s	40 cycles

**Table 2:** CC50, IC50 and TI corresponding to each incubation time calculated for Iodim and Ozodrop.

EYE DROP	INCUBATION TIME	CC50 (μL)	Antiviral Assay	IC50 (μL)	TI
Iodim	15 s	>100	Co-treatment	30	3.3
	30 s			25	4
	1 min			<12.5	<8
	10 min			<12.5	<8
	30 min			<12.5	<8
	1 h			<12.5	<8
Iodim	15 s		Virus pre-treatment	25	4
	30 s			12.5	8
	1 min			<12.5	<8
	10 min			<12.5	<8
	30 min			<12.5	<8
	1 h			<12.5	<8
Ozodrop	15 s	>100	Co-treatment	30	3.3
	30 s			25	4
	1 min			<12.5	<8
	10 min			<12.5	<8
	30 min			<12.5	<8
	1 h			<12.5	<8
Ozodrop	15 s		Virus pre-treatment	75	1.3
	30 s			25	4
	1 min			<12.5	<8
	10 min			<12.5	<8
	30 min			<12.5	<8
	1 h			<12.5	<8

## Data Availability

The data presented in this study are available on request from the corresponding author. The authors can confirm that all relevant data are included in the article.
